# A Literature Review of Glutathione Therapy in Ameliorating Hepatic Dysfunction in Non-Alcoholic Fatty Liver Disease

**DOI:** 10.3390/biomedicines13030644

**Published:** 2025-03-06

**Authors:** Michelle Thuy Nguyen, Andrew Lian, Frederick Timothy Guilford, Vishwanath Venketaraman

**Affiliations:** 1 College of Osteopathic Medicine of the Pacific, Western University of Health Sciences, Pomona, CA 91766, USA; michelle.nguyen2@westernu.edu (M.T.N.); andrew.lian@westernu.edu (A.L.); 2 Your Energy Systems, Palo Alto, CA 94301, USA

**Keywords:** nonalcoholic fatty liver disease or NAFLD, glutathione or GSH, oxidative stress, cellular damage, hepatic dysfunction, metabolic dysfunction

## Abstract

Non-alcoholic fatty liver disease (NAFLD) is a global cause of liver dysfunction. This spectrum of hepatic disorders can progress to severe conditions, such as non-alcoholic steatohepatitis (NASH) and cirrhosis, due to oxidative stress and sustained cellular injury. With limited pharmacological options, glutathione (GSH), a key antioxidant, has shown promising potential in reducing oxidative stress, maintaining redox balance, and improving liver function. This literature review examines studies from 2014–2024 exploring GSH therapy in NAFLD patients. Eligible studies assessed GSH as the primary intervention for NAFLD in human subjects, reporting outcomes such as liver function or oxidative stress markers. Randomized clinical trials (RCTs) were eligible, while combination therapy studies were included if GSH’s effect could be isolated. Exclusions applied to non-NAFLD studies, animal/in vitro models, and non-GSH antioxidant interventions. Analysis of three studies (totaling 109 participants) demonstrated consistent improvements in alanine transaminase (ALT) levels and reductions in oxidative stress markers like 8-hydroxy-2-deoxyguanosine (8-OHdG). However, small sample sizes and inconsistent protocols limit generalizability. Further large-scale RCTs are required to confirm GSH’s efficacy, determine optimal dosing, and assess long-term effects. This literature review highlights GSH’s potential as a novel NAFLD therapeutic strategy while emphasizing the need for further studies to refine its clinical application.

## 1. Introduction

**Figure 1:**
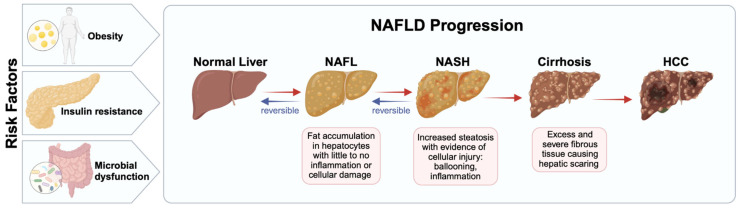
The progression of non-alcoholic fatty liver disease (NAFLD) follows a continuum, influenced by key risk factors such as microbial dysfunction, insulin resistance, and obesity. A healthy liver first develops into non-alcoholic fatty liver (NAFL) due to excessive fat accumulation, or steatosis. Without intervention, NAFL can advance to non-alcoholic steatohepatitis (NASH), characterized by worsening steatosis and cellular injury. As fibrosis progresses and remains untreated, NASH may evolve into cirrhosis—an irreversible stage of liver damage. Continued hepatic injury can ultimately lead to hepatocellular carcinoma (HCC), a severe and life-threatening complication.

Non-alcoholic fatty liver disease (NAFLD) is one of the most common chronic liver conditions worldwide, encompassing a spectrum of conditions characterized by varying degrees of hepatic steatosis and cellular injury [1,2]. It develops in the absence of secondary causes such as significant alcohol consumption, steatogenic medications, or other hepatic diseases [1,2]. NAFLD progresses in severity from simple steatosis, known as non-alcoholic fatty liver (NAFL), to non-alcoholic steatohepatitis (NASH), and ultimately to hepatic cirrhosis. This progression is driven by hepatocellular inflammation, damage, and subsequent fibrosis (Figure 1) [3]. Severe complications of this ongoing liver damage include hepatocellular carcinoma (HCC) and liver failure, often necessitating liver transplantation (LT) [3].

Younossi et al. reported the global prevalence of NAFLD to be approximately 25% between 1990 and 2015, with roughly 80 million people affected in the United States of America (U.S.A.) alone [4]. However, an updated study revealed a sharp increase, with the global prevalence of NAFLD rising to an astonishing 38% between 2016 and 2019 [5]. Additionally, NASH has become the fastest-rising culprit of HCC and is the second-leading cause of LTs in the U.S.A. [6,7,8,9]. Predictive modeling suggests that NAFLD cases will continue to rise, leading to increased liver-related mortality and complications worldwide from 2016 to 2030 [10,11].

The global prevalence of NAFLD has grown concurrently with the rise in obesity and type 2 diabetes mellitus (T2DM); however, there exist distinct geographical differences in NAFLD prevalence, disease burden, and severity across various countries [4,5,12,13]. In Asian countries, Riazi et al. found that Iran had the highest prevalence of NAFLD at 40.8% [13]. In contrast, Japan had a relatively low prevalence of NAFLD at 22.8%, which could be attributed to the country’s lower prevalence of obesity and a traditional diet that is naturally low in fat [13,14,15]. Furthermore, in a large cross-sectional study of 571,872 Korean military men in their early 20s, the prevalence of NAFLD increased significantly from 10.66% in 2015 to 16.44% in 2021 [16]. This rise paralleled a concurrent, significant increase in metabolic risk factors, including hypercholesterolemia, hyperglycemia, and hypertension [16]. In another study utilizing the Korea National Health and Nutrition Examination survey, NAFLD was seen to increase from 18.6% in 1998–2001 to 21.5% in 2016–2017 in conjunction with a rise in obesity and T2DM [17]. These findings suggest that the rising prevalence of NAFLD may be intrinsically linked to the worsening burden of metabolic risk factors. Of note, a hallmark of the NAFLD epidemic in Asia is the disproportionately high prevalence of lean NAFLD (body mass index [BMI] < 23) and non-obese NAFLD (BMI < 25), which highlights the unique metabolic susceptibilities in the region [12,18]. Asians are also more likely to develop T2DM at a younger age and with a lower BMI [19,20,21]. These regional differences not only influence NAFLD disease progression but also underscore the need for tailored therapeutic approaches that address the specific metabolic characteristics in Asian populations.

NAFLD prevalence in the U.S.A. has also increased alongside the obesity epidemic [13,22]. In a meta-analysis by Riazi et al., which examined 15,178 individuals from the U.S.A., the prevalence of NAFLD was estimated at 47.8% [13]. Another study found that Hispanics had the highest prevalence, followed by non-Hispanic Whites and non-Hispanic Blacks [23]. NAFLD also affects children, with a 62% rise in incidence in Southern California from 2009 to 2018 [24]. This trend is particularly concerning given that, over the past three decades, childhood obesity in the U.S. has more than doubled, while adolescent obesity has tripled [25]. The rapid rise in obesity strongly correlates with NAFLD trends, highlighting the urgent need for targeted interventions to curb metabolic risk factors across the region.

Currently, there is no standard treatment regimen for NAFLD [26]. Therapeutic approaches primarily emphasize mitigating disease progression through lifestyle interventions, including weight management and alcohol abstinence. Pharmacological treatments are generally reserved for individuals with advanced stages of NAFLD and aim to manage comorbid conditions such as hypertension, dyslipidemia, and diabetes, which can exacerbate liver dysfunction [26]. The absence of effective therapies, coupled with the ongoing challenges of addressing underlying factors, is projected to increase the number of individuals affected by NAFLD in the U.S.A. to 100 million by 2030 [3,6,11,27,28].

Given the limited therapeutic options and the growing global prevalence of NAFLD, this literature review examines the efficacy of glutathione (GSH)—a tripeptide crucial for reducing oxidative stress, maintaining redox balance, and modulating immune responses—in improving liver function in patients with NAFLD [4,5,29]. Animal studies utilizing rodent models have shown that GSH supplementation can attenuate liver injury and enhance the degradation of unwanted cellular materials, making it a compelling therapeutic option for mitigating NAFLD disease progression [30,31,32,33]. This paper has therefore chosen to focus specifically on GSH therapy in human trials as a potential treatment for this disease.

In recent years, the term metabolic dysfunction-associated steatotic liver disease (MASLD) has been proposed to replace NAFLD, emphasizing its role as a hepatic manifestation of broader systemic metabolic dysregulation [34]. Similar to NAFLD, MASLD is characterized by hepatic steatosis exceeding 5% but incorporates additional diagnostic criteria linked to cardiometabolic dysfunction, including obesity (defined by body mass index), dyslipidemia, and T2DM [4,34,35]. This revised nomenclature better reflects the systemic and metabolic dysfunctions that influence disease progression [36]. While it does not directly alter the fundamental understanding of GSH therapy, it may encourage more targeted investigations into how GSH interacts with specific metabolic pathways and patient subgroups, as well as its effects on NAFLD risk factors and comorbidities. However, given the continued popularity of the term NALFD, we will refer to the condition as such throughout the remainder of this paper.

## 2. Pathogenesis of Non-Alcoholic Fatty Liver Disease

The pathogenesis of NAFLD is multifactorial, involving complex interactions between genetic, metabolic, and environmental factors. Initially, the pathogenesis of NASH was explained by the “2-hit hypothesis”, where the first hit, hepatic triglyceride accumulation (steatosis), increases the liver’s susceptibility to injury from second hits, such as inflammatory cytokines, mitochondrial dysfunction, and oxidative stress [37,38]. This progression ultimately leads to steatohepatitis and fibrosis. However, recent advancements now highlight the role of free fatty acids (FFAs) in facilitating liver injury, calling for a modification to the theory.

Insulin resistance plays a central role in NAFLD development by increasing lipolysis in adipose tissue, leading to an accumulation of FFAs in the liver [39,40]. Excessive FFAs promote mitochondrial β-oxidation and peroxisomal fatty acid metabolism, generating excessive reactive oxygen species (ROS) [41]. This oxidative stress, in turn, contributes to hepatocyte injury, inflammation, and the progression of liver damage [40,42]. Additionally, mitochondrial dysfunction and oxidative stress play crucial roles in the pathogenesis of NAFLD. Mitochondria in hepatocytes are key to lipid metabolism, fatty acid oxidation, and energy production. Their dysfunction leads to increased oxidative stress and ROS production due to exhaustion of cellular antioxidative capacity [43,44,45]. This oxidative stress triggers lipid peroxidation, where ROS react with polyunsaturated fatty acids in cell membranes, forming reactive aldehydes such as malondialdehyde (MDA) and 4-hydroxynonenal (4-HNE) [46,47,48]. These aldehydes, which have been shown to be increased in NAFLD patients and are used as biomarkers of lipid peroxidation in clinical practice, further damage cellular structures and promote inflammation [46,47,48,49]. By altering fluidity and permeability, lipid peroxidation compromises the cellular membrane and significantly disrupts overall cellular function [48,50]. Furthermore, ferroptosis, a regulated cell death pathway driven by iron-dependent lipid peroxidation, has recently emerged as a key mechanism in NAFLD progression [51,52]. Elevated iron levels, which are commonly observed in NAFLD patients, catalyze ROS formation via the Fenton reaction [53]. This thereby accelerates lipid peroxidation and hepatocyte death, ultimately contributing to hepatic inflammation and fibrosis [53].

Gut microbial dysbiosis has also been implicated in the pathogenesis of NAFLD. The gut microbiome consists of diverse microbial communities that interact with one another and the human body, significantly influencing overall human health [54]. Central to this interaction is the gut–liver axis, a bidirectional communication network between the gut microbiota and the liver, which is essential for maintaining systemic homeostasis [55,56]. In individuals with NAFLD, microbial shifts disrupt this balance, leading to dysbiosis [56,57,58,59,60,61]. Studies have shown that NAFLD is associated with an increased abundance of pro-inflammatory bacterial products, which can compromise gut barrier integrity and increase intestinal permeability [57,58,60,61]. This allows bacterial endotoxins, such as lipopolysaccharides, to enter the portal circulation, triggering hepatic inflammation and contributing to liver injury [62,63]. Additionally, alterations in microbial composition impact bile acid metabolism and short-chain fatty acid production, further influencing lipid metabolism and insulin sensitivity in the liver [55,56]. As a result, gut dysbiosis not only contributes to liver injury but also reinforces the metabolic dysfunction underlying NAFLD, making it a key factor in disease development and progression.

Impaired insulin signaling, diminished mitochondrial oxidative capacity, lipid peroxidation, ferroptosis, and microbial dysbiosis not only exacerbate hepatic steatosis but also facilitate the progression from simple steatosis to more severe forms of the disease, such as NASH and cirrhosis [51,56,63,64,65,66,67]. GSH plays a key role in reducing lipid peroxides, preventing oxidative damage that may trigger ferroptosis, and maintaining cellular redox balance, thereby mitigating lipid peroxidation and oxidative stress in NAFLD [51,68]. Overall, modulating the drivers of oxidative stress and cellular dysfunction offers a promising avenue for halting or reversing the progression of NAFLD.

## 3. Glutathione Background

It is well established that oxidative stress plays a key role in driving NAFLD pathogenesis [44,45]. The imbalance between pro-oxidants and antioxidants leads to lipid peroxidation, mitochondrial dysfunction, and inflammation, all of which contribute to hepatocellular damage [43,46,48,49]. Additionally, risk factors such as obesity, T2DM, chronic inflammation, and gut microbiota dysbiosis exacerbate oxidative stress, further promoting disease progression (Figure 1) [3,17,63]. Given the central role of oxidative stress in NAFLD, antioxidant therapies have been explored as potential treatments. Various antioxidants, such as vitamin E and resveratrol, have been studied for their hepatoprotective properties [69,70]. However, their efficacy remains inconsistent, and concerns regarding long-term safety have limited their widespread use [71,72].

Resveratrol, a natural compound found in grapes, possesses anti-inflammatory and antioxidant properties [69,73,74]. It can also influence microbial composition by promoting the growth of beneficial bacteria while suppressing harmful species, ultimately enhancing gut barrier function and reducing gut dysbiosis [69]. However, resveratrol has been reported to inhibit cell growth and induce apoptosis in normal cells at high doses, with studies showing that mice consuming large doses experienced fatal outcomes within 3–4 months [75,76,77,78]. Vitamin E has also been widely considered as a potential treatment for liver disease because of its function as a potent free radical scavenger [79]. Long-term vitamin E therapy (300 mg/day for over two years) has been shown to improve hepatic fibrosis in NASH patients, particularly those with reduced serum transaminase levels, and insulin resistance [80]. However, some studies suggest that vitamin E treatment may increase risks of all-cause mortality and hemorrhagic stroke, though conflicting results make these studies inconclusive [72,81].

Among antioxidant therapies, GSH has emerged as a promising candidate due to its critical role in cellular detoxification and redox balance [29]. GSH, or γ-L-glutamyl-L-cysteinyl-glycine, is a tripeptide synthesized in the liver. The liver serves as the primary source of plasma GSH, thereby playing a crucial role in maintaining interorgan GSH homeostasis; thus, hepatic dysfunction has a systemic impact on oxidative stress and redox balance [29,82,83,84,85,86]. Approximately 80–85% of GSH is stored in the cytosol, while 10–15% resides in the mitochondria, where its concentration is balanced through specific transport mechanisms between the matrix and cytosol [29,83,84].

**Figure 2:**
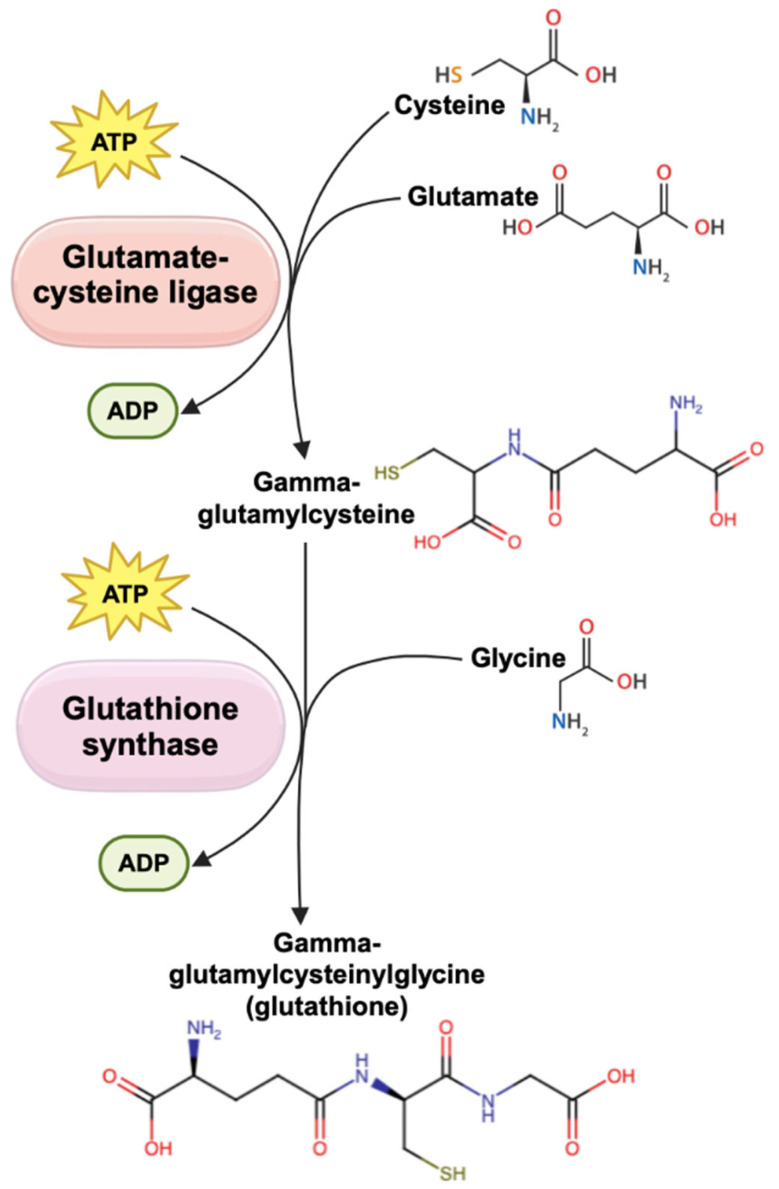
The synthesis of glutathione (GSH), also known as γ-glutamylcysteinylglycine, occurs through an ATP-dependent enzymatic process essential for maintaining cellular redox balance. In the first step, glutamate-cysteine ligase (GCL) catalyzes the rate-limiting conjugation of glutamate and cysteine, forming γ-glutamylcysteine. In the second step, GSH synthase (GS) facilitates the addition of glycine, completing the formation of GSH.

In a process called the γ-glutamyl cycle, GSH is continuously recycled to ensure a steady-state supply in cells. The process begins with the extracellular breakdown of GSH into its constituent amino acids—glutamate and cysteine—by hepatic γ-glutamyl transferase (GGT), an enzyme present only on the external surfaces of cell types, such as those of the hepatobiliary tree and heart [87,88]. These amino acids are then transported into the cell for the next step [89]. Between these amino acids, cysteine availability is the rate-limiting factor in GSH biosynthesis [83,90]. Extracellular cysteine is relatively unstable and readily autoxidizes to cystine, which can lead to the production of toxic oxygen free radicals [83,87,91]. Due to its instability, the cystine/glutamate antiporter (xCT) plays a pivotal role in cystine uptake [91,92,93]. The xCT exchanges intracellular glutamate for extracellular cystine, which is reduced to cysteine in the cytosol and ready for GSH synthesis [82,92,93,94,95]. Additionally, in a separate metabolic pathway known as the transsulfuration (cystathionine) pathway, methionine can be metabolized in the liver to provide an alternative source of cysteine for GSH production [83,96,97]. GSH can then be synthesized de novo in a two-step ATP-requiring enzymatic process utilizing glutamate cysteine ligase (GCL) and glutathione synthetase (GS) (Figure 2) [83,87]. GCL catalyzes the formation of γ-glutamylcysteine from glutamate and cysteine, which is then combined with glycine via GS to produce GSH [83,88]. This process, coupled with GSH utilization and reduction, constitutes the γ-glutamyl cycle, a process that ensures a steady state of GSH in cells [88].

GSH primarily exists in two states: the reduced form (GSH), which is the dominant state, and the oxidized form, glutathione disulfide (GSSG), where two GSH molecules are connected via a disulfide bond [29,87]. The ratio of GSH to GSSG is an indicator of the redox status of cells. In healthy resting cells, this ratio exceeds 100, but it decreases to a range of 1 to 10 when cells are subjected to oxidative stress [29,83,98]. During these periods of oxidative stress, levels of GSSG may rise, prompting the induction of GGT to facilitate the recycling of precursor amino acids for GSH synthesis to maintain a balanced GSH-to-GSSG ratio [29,87]. Additionally, the accumulation of GSSG and depletion of GSH can be directly toxic to cells by activating the SAPK/MAPK apoptotic signaling pathway, which leads to programmed cell death [29,83,98,99]. The balance of GSH is, therefore, significant to the maintenance of cellular homeostasis. As a result, GGT serves not only as a marker of oxidative stress but also as a critical component of the antioxidant defense system, making it particularly valuable in the context of NAFLD.

**Figure 3:**
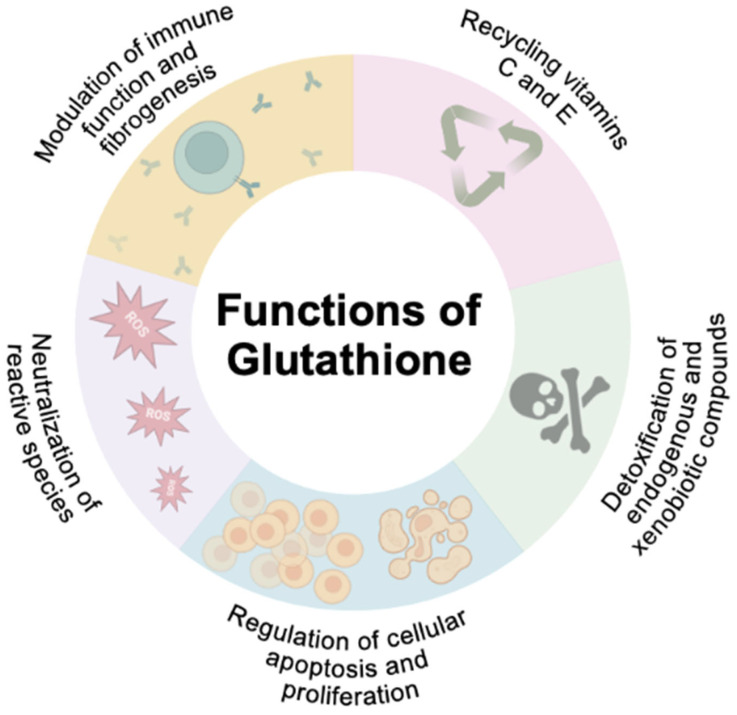
Glutathione (GSH), a powerful intracellular antioxidant, plays a crucial role in maintaining cellular homeostasis by neutralizing oxidative stress, preventing lipid peroxidation, and protecting cells from free radical-induced damage. As a central component of the body’s detoxification system, GSH supports the elimination of toxins, enhances mitochondrial function, and regulates immune responses. Its ability to modulate redox balance is essential for preserving cellular integrity, promoting metabolic efficiency, and reducing the risk of chronic diseases associated with oxidative stress.

GSH additionally plays a multifaceted role in cellular defense, primarily by reducing oxidative stress and facilitating detoxification (Figure 3). It directly scavenges ROS and reactive nitrogen species (RNS), including superoxide anions, hydroxyl radicals, nitric oxide, and carbon radicals [29,84,87]. Additionally, it catalytically detoxifies harmful compounds such as hydroperoxides, peroxynitrites, and lipid peroxides, protecting cells from damage caused by these reactive molecules [29,87]. Without GSH, these toxic species may induce hepatocellular stress, injury, and death, leading to hepatic fibrogenesis and genomic instability that predispose to cirrhosis and HCC [3]. GSH also supports the regeneration of key antioxidants, such as vitamins C and E, thereby enhancing their effectiveness in combating oxidative stress (Figure 3) [29].

Beyond its antioxidant and detoxifying properties, GSH is essential for maintaining physiological balance. GSH plays a pivotal role in maintaining redox balance, regulating immune responses, and supporting energy production and cellular repair (Figure 3) [29]. However, under oxidative stress, the depletion of GSH and accumulation of GSSG can compromise these functions, triggering apoptotic pathways and contributing to cell death [29,84]. This underscores the vital role of GSH in maintaining cellular homeostasis and its potential therapeutic importance in conditions characterized by oxidative damage.

This therapeutic potential of GSH has sparked significant interest in its potential therapeutic role in managing various chronic diseases associated with altered redox balance and decreased GSH levels. Notably, cellular and mitochondrial GSH levels are strongly correlated with health and longevity, underscoring its critical importance in preventing and mitigating diseases associated with GSH depletion [29]. Conditions such as neurodegenerative disorders, cancer, and liver diseases such as NAFLD have been closely linked to GSH homeostasis disruptions [29,83,87]. Because a majority of plasma GSH is synthesized from the liver, hepatic impairment has a systemic impact on redox balance and oxidative stress [29,83,84]. This emphasizes the critical role of GSH in mitigating cellular damage, slowing disease progression, and supporting overall liver health. The capacity of GSH to counteract oxidative stress and regulate cellular homeostasis positions it as a promising therapeutic target for a wide range of oxidative stress-related diseases [29,83,84,87].

## 4. Methods

A literature search was conducted to identify studies examining the therapeutic potential of GSH for NAFLD in human models. The search was carried out through electronic databases PubMed, Google Scholar, and ScienceDirect, covering studies published between 2014 and 2024. Search terms such as “glutathione”, “non-alcoholic fatty liver disease”, “NAFLD”, and “oxidative stress” were used, with Boolean operators to combine terms. Filters were applied to include only peer-reviewed articles in English. To ensure comprehensive coverage, reference lists from relevant articles were manually screened for additional studies.

Studies were included if they met predefined eligibility criteria: (1) they investigated GSH as the primary intervention for NAFLD; (2) they were conducted in human subjects; and (3) they reported relevant outcome measures, such as changes in liver function tests, e.g., alanine transaminase (ALT), aspartate aminotransferase (AST), GGT levels, histological improvements in liver tissue, or reductions in oxidative stress biomarkers such as 8-hydroxy-2-deoxyguanosine (8-OHdG). Both randomized controlled trials (RCTs) and observational studies were considered eligible, provided they included sufficient quantitative data for review. Studies involving combinations of pharmacological therapies were included only if the effect of GSH could be isolated.

Exclusion criteria included studies that focused on general liver disease populations without NAFLD-specific data. This decision was made because liver diseases vary significantly in their reversibility, mechanisms, and treatment responses. Including such studies could lead to misleading conclusions about GSH’s efficacy, as its potential benefits in NAFLD may not apply to more advanced or non-metabolic liver diseases. As such, it is important to focus on studies primarily evaluating NAFLD to ensure proper treatment response with GSH is obtained. Other exclusion criteria included animal or in vitro models and antioxidant interventions other than GSH. Case reports, editorials, conference abstracts, and reviews were also excluded to maintain a focus on primary research. Articles that did not provide measurable outcomes or lacked full-text availability were also omitted, with corresponding authors contacted, when possible, to obtain missing data.

The initial search yielded 12 articles, which were screened for duplicates and relevance based on titles and abstracts. Full-text screening was subsequently performed for these 12 articles, with 3 studies meeting the inclusion criteria. Data extraction was conducted independently by two reviewers using a standardized data collection form, capturing details such as study design, sample size, participant characteristics, GSH dosage and duration, and reported outcomes. Any discrepancies between reviewers were resolved through discussion.

## 5. Results

This review included three studies with a total of 109 participants (Table 1) [100,101,102]. Treatment with GSH demonstrated improvements in ALT levels across all studies in patients with NAFLD [100,101,102]. However, statistically significant reductions in ALT (*p* < 0.05) were observed only in the studies by Honda et al. and Irie et al. [101,102]. The study performed by Reddy et al. did not indicate *p*-value comparison within treatment groups before and after GSH intervention; therefore, the significance of ALT change in addition to GGT is undetermined [100]. This limitation hinders a precise assessment of GSH’s direct efficacy, emphasizing the need for more research studies with comprehensive statistical evaluations.

In Irie et al.’s study, the improvement in ALT was observed exclusively in the group of patients with NASH [102]. These patients were also observed to have significant decreases in 8-OHdG (*p* < 0.05) following GSH treatment [102]. Improvements in ALT levels suggest better liver function, as lower ALT levels indicate reduced liver cell damage and inflammation [103,104]. Similarly, reductions in 8-OHdG, a marker of DNA damage from oxidative stress, suggest a decrease in oxidative damage [105,106,107]. Together, these improvements indicate better liver health. Conversely, those with NASH exhibited nonsignificant improvements in GGT, and patients with NAFL demonstrated nonsignificant improvements in ALT, GGT, and 8-OHdG after treatment [102]. This perhaps could be due to the fact that NASH is a more severe form of NAFLD compared to NAFL, leading to more pronounced effects of GSH treatment. The greater severity of liver damage in NASH may make improvements more detectable and statistically significant. Additionally, only five patients with NAFL were evaluated in the study, making the sample size too small to draw definitive conclusions about the effects of GSH on liver function in cases of simple fatty liver.

In Honda et al.’s study, patients adhered to an exercise and diet regimen prior to the initiation of GSH therapy. Given that lifestyle modifications, including dietary adjustments and physical activity, are well-established interventions for improving liver function in patients with NAFLD, these factors may have positively contributed to the observed outcomes [108,109,110]. Consequently, it is essential to distinguish the independent effects of GSH from those of lifestyle modifications when assessing its true therapeutic efficacy. Furthermore, this study did not differentiate between NAFL and NASH within its patient cohort. Evaluating the efficacy of GSH across varying stages of liver dysfunction could offer valuable insights into its therapeutic potential, guiding optimal treatment durations and dosing strategies.

Additionally, in the study by Honda et al., liver fat was non-invasively assessed using vibration-controlled transient elastography (VCTE) with a controlled attenuation parameter (CAP), a method that has been shown to have good sensitivity and specificity in detecting hepatic steatosis [101,111]. Although nonsignificant, treatment with GSH showed improvements in both CAP values and liver stiffness measurements (LSM). However, further research is required to draw definitive conclusions.

All three studies were conducted in Asian countries (Japan and India) with exclusively Asian participants, limiting the generalizability of the findings. Given that NAFLD presents at a lower BMI and younger age in Asian populations compared to other ethnic groups, the effects of GSH observed in these studies may not be directly applicable to broader, more diverse populations [19,20,21]. Ethnic variations in NAFLD progression underscore the need for further research across different ethnicities and geographical regions. Conducting studies in diverse populations will be crucial to establishing the efficacy of GSH in a more universally representative manner and drawing robust, generalizable conclusions.

Although all three studies demonstrated overall improvements in ALT levels, suggesting an improvement in liver function, variations in statistical significance and differences across NAFLD stages underscore the need for more comprehensive research to better understand GSH’s efficacy and its impact on different disease severities. Additionally, future studies should include larger patient populations, a broader range of liver pathologies, and diverse ethnic groups to ensure more generalizable findings and to account for potential variations in treatment response based on these factors. This would provide a more nuanced understanding of GSH’s therapeutic potential in various patient subgroups.

## 6. Discussion

**Figure 4:**
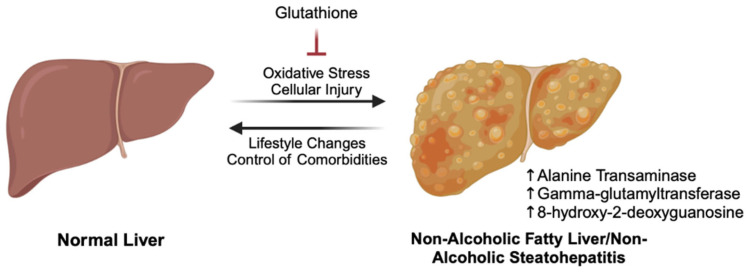
Glutathione (GSH) targets key drivers of NAFLD by mitigating oxidative stress and cellular injury, which are central to inflammation and hepatic dysfunction. This helps improve liver function and modulate biomarkers of liver health, including alanine transaminase (ALT) and γ-glutamyltransferase (GGT), indicators of liver function and oxidative stress, as well as 8-hydroxy-2-deoxyguanosine (8-OHdG), a marker of oxidative DNA damage. Its protective effects may contribute to slowing disease progression and improving overall metabolic health.

The results of this literature review suggest that GSH may have therapeutic potential in the management of NAFLD, particularly for improving liver function and reducing oxidative stress (Figure 4). Among the three included studies, ALT levels consistently improved, highlighting GSH’s potential in reducing hepatic inflammation and injury in NAFLD patients [100,101,102]. Along with ALT reductions, oxidative stress markers like GGT and 8-OHdG also decreased—key indicators of oxidative damage and inflammation in NAFLD progression [29,84,112]. However, the significance of findings varies across studies. Irie et al. reported significant improvements in ALT and 8-OHdG only in NASH patients, while the NAFL group showed only nonsignificant reductions [102]. Irie et al.’s study underscores the potential variability of GSH’s effects across different NAFLD stages, possibly due to the greater severity of liver damage in NASH compared to NAFL. Although Honda et al. reported significant reductions in ALT and Reddy et al. reported reductions of uncertain significance, these studies did not distinguish between NAFLD stages [100,101]. To better understand GSH’s impact on hepatic function and oxidative stress, future studies should clearly define NAFLD stages among participants to accurately assess its effects.

Despite promising findings of GSH, several limitations must be acknowledged when interpreting this analysis. First, the small number of included studies and the relatively modest sample size of 109 participants limit the generalizability of the results [100,101,102]. Additionally, as the study populations were restricted to participants from India and Japan, the applicability of these findings to other ethnic groups remains uncertain due to genetic, dietary, and lifestyle differences that influence NAFLD progression and treatment response [100,101,102]. For instance, variations in the prevalence of genetic polymorphisms such as PNPLA3 and TM6SF2, both strongly associated with NAFLD susceptibility and progression in Chinese populations, may impact the effectiveness of GSH treatment [113,114,115,116]. Furthermore, heterogeneity in NAFLD severity, comorbid conditions, and baseline metabolic profiles complicates direct comparisons across studies. The interplay of these variables underscores the need for larger, multicenter trials with more ethnically diverse populations to better elucidate the role of GSH in NAFLD management. Expanding the scope of research will enhance the external validity and clinical applicability of these findings to a broader patient demographic.

Moreover, while two studies reported significant findings, the inconsistency in statistical significance across all studies highlights the need for large-scale, multicenter, well-powered RCTs to confirm these results [100,101,102]. Another critical limitation is the absence of control groups, as all evaluated studies were open-label, single-arm trials [100,101,102]. Without a placebo or comparative treatment group, it remains unclear whether the observed improvements in hepatic function were directly attributable to GSH therapy or influenced by external factors such as lifestyle modifications, concurrent medications, or the natural course of NAFLD progression. These methodological constraints underscore the necessity for rigorously designed trials with well-defined patient stratification, standardized treatment protocols, and long-term follow-up to elucidate GSH’s true therapeutic potential in NAFLD management.

Another notable limitation is the potential risk of bias in the included studies. Although quality assessments were conducted, the lack of uniform blinding and randomization methods could introduce bias in outcome reporting. Furthermore, the focus on ALT and oxidative stress markers provides a limited perspective, as other critical outcomes, such as liver histology, fibrosis staging, and metabolic parameters, were not consistently evaluated, limiting a comprehensive assessment of GSH’s therapeutic potential. Metabolic parameters—including insulin resistance, lipid profiles, and gut microbiome alterations—play a significant role in NAFLD pathogenesis and could provide a broader understanding of GSH’s systemic effects. Furthermore, elucidating the interplay between GSH and critical molecular pathways, including mitochondrial function and lipid metabolism, could provide valuable mechanistic insights and help identify more biomarkers predictive of treatment response. Future research should adopt a more comprehensive approach by incorporating these additional endpoints into well-designed RCTs and examining the long-term effects of GSH supplementation on liver histology.

Based on the currently available research, GSH dosage, duration, and administration were consistent across all surveyed studies, with a daily oral dose of 300 mg [100,101,102]. However, a notable difference in the protocols was noted among the studies. Honda et al.’s study incorporated lifestyle interventions such as diet and exercise alongside GSH, which introduces confounding variables that obscure the potential independent effects of GSH on liver function [101]. Therefore, to isolate the specific contribution of GSH to NAFLD treatment, future trials must control for lifestyle factors or clearly delineate the interactions between these interventions and GSH administration. Additionally, investigating the potential synergy between GSH and other therapeutic approaches, such as lifestyle interventions or anti-inflammatory agents, may enhance its efficacy and warrants additional investigation.

Another important consideration when interpreting the findings of this review is the evolving nomenclature and diagnostic criteria for MASLD, which has replaced the term NAFLD in recent years [4,34,35]. This reclassification represents more than just a terminological update; it reflects a growing recognition of the central role of metabolic dysfunction in the pathogenesis of steatotic liver disease and aligns diagnostic criteria with our expanding understanding of its underlying mechanisms. The shift to MASLD aims to provide greater clarity in disease characterization, distinguishing it from other causes of liver fat accumulation while emphasizing its strong association with metabolic comorbidities, such as obesity, insulin resistance, dyslipidemia, and T2DM. While the studies included in this review were conducted under the NAFLD classification, the transition to MASLD provides a more precise framework for stratifying and managing patients with coexisting metabolic disorders [117]. This, in turn, could influence the selection of study populations in clinical trials and improve the generalizability of findings to real-world patient cohorts. Given GSH’s role in mitigating oxidative stress, its therapeutic potential may vary across various metabolic profiles of MASLD subgroups. As a result, therapeutic interventions targeting metabolic pathways, including antioxidants such as GSH, may require reassessment in light of these evolving definitions. Under the MASLD classification, it is also important to evaluate how GSH influences not only hepatic oxidative damage but also the broader metabolic drivers of disease. Future research should explore whether GSH supplementation can modulate these interconnected pathways. For example, investigating the impact of GSH on insulin sensitivity, lipid metabolism, and pro-inflammatory cytokine levels could provide valuable insights into its role as a metabolic therapy. Furthermore, the transition to MASLD highlights the need for a more nuanced approach to treatment, moving beyond a liver-centric perspective to consider systemic metabolic health. If GSH proves beneficial in mitigating metabolic dysfunction—by improving insulin sensitivity, reducing inflammation, or enhancing mitochondrial efficiency—it could be a valuable adjunct therapy in MASLD management. By reassessing the effects of GSH within the context of MASLD’s metabolic underpinnings, investigators can generate findings that are more applicable to contemporary diagnostic and therapeutic frameworks. This will facilitate the development of more targeted and effective management strategies, ensuring that interventions are optimally tailored to the metabolic and hepatic disease profiles of MASLD patients.

Overall, this literature review provides a focused synthesis of the existing research on GSH therapy for hepatic dysfunction in NAFLD, highlighting both its potential benefits and the critical gaps in current knowledge. By compiling and analyzing the available studies, we offer a clearer understanding of GSH’s proposed mechanisms—primarily its role in reducing oxidative stress and modulating inflammatory pathways. This focused approach provides a valuable reference for clinicians and researchers seeking to contextualize GSH within the broader spectrum of NAFLD intervention. Additionally, a key contribution of this review is its assessment of methodological limitations in the current literature. Most studies analyzed were single-arm, open-label investigations, underscoring the absence of RCTs necessary to establish definitive efficacy. This lack of rigorous study design raises concerns regarding bias, placebo effects, and the inability to compare GSH with standard treatments or alternative antioxidant therapies. Furthermore, the geographic concentration of existing studies, primarily in Asian populations, highlights the need for broader, more diverse cohorts to improve generalizability. Ultimately, this review underscores the pressing need for well-designed, multicenter RCTs with standardized outcome measures and diverse patient populations to determine the true clinical utility of GSH in NAFLD management. Addressing these gaps will be essential to validating GSH as a viable therapeutic option and guiding its integration into future treatment paradigms.

## 7. Conclusions

This review underscores the potential therapeutic role of GSH in the treatment of NAFLD in humans. The included studies consistently demonstrate improvements in liver function parameters and reductions in oxidative stress markers following GSH treatment intervention. These findings align with the established role of oxidative stress in the pathogenesis of NAFLD and suggest that GSH may help mitigate liver damage by enhancing antioxidant defenses. However, the heterogeneity in study quality and the limited number of high-quality studies highlight the preliminary nature of these results.

Given these limitations, further research is needed to confirm the efficacy and optimal dosing strategies of GSH interventions in diverse populations. Future studies should involve an increased number of patients with varying degrees and stages of liver dysfunction, as this would provide a more comprehensive understanding of how GSH impacts liver health across the spectrum of NAFLD. Additionally, to establish GSH as a reliable therapeutic option, future studies should prioritize well-designed, high-quality RCTs with standardized protocols to ensure consistency in treatment approaches and facilitate robust comparisons across different populations and ethnicities.

While the current evidence points to the potential of GSH as a therapeutic intervention for NAFLD, these findings should be interpreted with caution, and clinical recommendations should await further validation. Despite these challenges, the overall findings suggest that GSH holds promise as a therapeutic intervention for NAFLD by targeting oxidative stress pathways and improving liver health outcomes.

## Figures and Tables

**Table 1:** Studies analyzed in this review. 8-OHdG: 8-hydroxy-2-deoxyguanosine, ALT: alanine transaminase, ALP: alkaline phosphatase, AST: aspartate transaminase, CAP: controlled attenuation parameter, GSH: glutathione, GGT: gamma-glutamyl transpeptidase, LSM: liver stiffness measurement, Tbili: total bilirubin.

Study and Year	Country	Type of Study	Population	Treatment	Outcomes of Measurement	Time Points of Measurement	Results
Irie [102], 2016	Japan	Open label, single arm	10 patients with NASH; 5 patients with fatty liver	300 mg of glutathione orally per day for 3 months	ALT, 8-OHdG, GGT	1 and 3 months	Significant (*p* < 0.05) ↓ ALT and 8-OHdG in NASH after treatment. Significant (*p* < 0.05) ↑ GSH levels in NASH after treatment. Nonsignificant ↓ GGT in NASH after treatment. Nonsignificant ↓ ALT, GGT, and 8-OHdG in NAFL after treatment.
Honda [101], 2017	Japan	Open label, single arm	34 patients with NAFLD	Standard diet (30 kcal/kg/day, 50–60% carbohydrate, 20–30% fat, 15–20% protein) and exercise (5–6 metabolic equivalents for 30 min daily) for 3 months followed by 300 mg of glutathione orally per day for 4 months	ALT, CAP, LSM	4 months	Significant (*p* < 0.05) ↓ ALT. Nonsignificant improvement in CAP and LSM.
Reddy [100], 2020	India	Open label, single arm	40 patients with NAFLD; 20 patients with fatty liver	300 mg of glutathione orally per day for 3 months	ALT, ALP, AST, Tbili, GGT, 8-OHdG	1 and 3 months	Study did not indicate *p*-value comparison within treatment groups; therefore, Undetermined significance of ↓ ALT and GGT in both groups after treatment. Significant (*p* < 0.01) ↑ GSH levels in both groups after treatment.

## Data Availability

No new data were created or analyzed in this study. Data sharing is not applicable to this article.
